# *Streptococcus suis* serotype 9 endocarditis and subsequent severe meningitis in a growing pig despite specific bactericidal humoral immunity

**DOI:** 10.1099/jmmcr.0.005093

**Published:** 2017-05-03

**Authors:** Karoline Rieckmann, Kristin Müller, Annette Moter, Christoph G. Baums, Anna Seydel

**Affiliations:** ^1^​ Institute for Bacteriology and Mycology, Centre for Infectious Diseases, Faculty of Veterinary Medicine, University Leipzig, Germany; ^2^​ Institute of Pathology, Faculty of Veterinary Medicine, University Leipzig, Germany; ^3^​ Biofilmcenter, German Heart Institute Berlin, Germany

**Keywords:** *Streptococcus suis*, endocarditis, meningitis, biofilm

## Abstract

**Introduction:**

. Meningitis and endocarditis are common pathologies of *Streptococcus* *suis* infections in pigs and humans. *S. suis* serotype 9 strains contribute substantially to health problems in European pig production, and immune prophylaxis against this serotype is very difficult.

**Case presentation:**

. We report the clinical course and histopathological picture of a 10-week-old growing pig following experimental intravenous infection with *S. suis* serotype 9. The piglet showed rapid onset of severe clinical signs of meningitis 11 days post-intravenous challenge following prime-booster vaccination. Histopathological findings revealed a diffuse fibrinosuppurative leptomeningitis. Additionally, a polyphasic *endocarditis valvularis thromboticans* with numerous bacterial colonies was diagnosed. Bacteriological culture of the brain and the mitral valve confirmed association with the challenge strain. However, virulent serotype 2 and 9 strains were killed in the blood of this piglet *ex vivo* prior experimental infection.

**Conclusion:**

. Endocarditis induced by *S. suis* infection might develop and persist despite the presence of high specific bactericidal activity in the blood. Severe leptomeningitis is a putative sequela of such an endocarditis.

## Abbreviations

DAPI, 4',6-diamidino-2-phenylindole; FISH, fluorescence in situ hybridization; MRP, muramidase-released protein.

## Introduction

*Streptococcus* *suis* is an invasive porcine pathogen and responsible for high economic losses in the pig industry worldwide. In swine *S. suis* causes severe pathologies like meningitis, arthritis, endocarditis and septicaemia [1]. While serotype 2 strains are worldwide most prevalent among porcine isolates, serotype 9 is also an important serotype in Europe [2–4]. In particular, serotype 9 contributes substantially to *S. suis*-associated invasive diseases of piglets in some areas with an unusually large number of piggeries like Lower Saxony, Germany.

Moreover, as an emerging zoonotic agent, *S. suis* is responsible for human infections mainly causing meningitis, septicaemia and streptococcal toxic shock-like syndrome [5–7]. The number of reported human cases has dramatically increased in recent years [7], and besides human infections with *S. suis* serotype 2, cases of several other serotypes causing disease in humans were described [5, 7–9]. Recently, the first case of a *S. suis* serotype 9 infection in a human patient was published [10].

The case described here shows that *S. suis* serotype 9 survives in vegetation on an atrioventricular valve despite specific bactericidal activity in the blood. The report reminds veterinarians and physicians that a polyphasic endocarditis is a risk for sudden onset of severe complications associated with central nervous system dysfunction.

## Case Report

As part of an experimental challenge study with vaccinated and placebo-treated pigs from a herd known to be free of *S. suis* serotype 9, a healthy, vaccinated German Landrace piglet (No. 346) was intravenously infected with 4×10^8^ and 1.4×10^9^ c.f.u. of a muramidase-released protein (MRP)- and suilysin-positive *S. suis* serotype 9 strain (A3286/94) at an age of 64 and 67 days, respectively. Infection doses were in the range of those described previously [11, 12]. We chose a higher dose in the second infection because in contrast to our previous trials no specific clinical signs were observed in the placebo-treated and vaccinated piglets in the two days following the first infection. Strain A3286/94 is a sequence type 99 strain belonging to clonal complex 16, and was originally isolated from a pig with meningitis [11].

Before challenge, the piglet was prime- and booster-vaccinated with recombinant immunoglobulin M-degrading enzyme of *S. suis* (rIde*_Ssuis_*) as previously described [13]. Prior to infection every twelve hours, and post-infection every eight hours, the piglet was monitored, recording parameters like inner body temperature, appetite, behaviour and movement. One day after the first experimental infection, the piglet showed a short non-recurring increase in body temperature to 40.1 °C without any other signs of illness. In the following days, the piglet remained clinically unobtrusive until the 11th day post-infection when signs of severe acute disease occurred. Specifically, the piglet had an inner body temperature of 41.3 °C along with faintness and anorexia. Furthermore, it showed a profound tremor in several body parts as well as acute kyphosis and neck stiffness with pain vocalization. For animal welfare reasons, the piglet was immediately anaesthetized by intramuscular application of 2 mg azaperone and 10 mg ketamine hydrochloride per kg body weight. After occurrence of anaesthesia, the piglet was euthanized through intravenous application of 90 mg pentobarbital per kg body weight. A comprehensive necropsy was subsequently conducted.

## Investigations

The immune status of the piglet prior and post experimental infection was analysed using ELISAs measuring serum IgG antibodies against MRP and Ide*_Ssuis_* as described [11, 12]. A serum from a piglet immunized with rIde*_Ssuis_* served as reference serum containing 100 ELISA units per definition. A convalescent-phase serum obtained from a piglet 20 days after experimental infection with *S. suis* serotype 2 strain 10 was used as a standard for the MRP ELISA (also defined to include 100 ELISA units).

The doses in the experimental infections and the specific bacterial loads of the samples of the bactericidal assays were determined by serial dilutions, plating appropriate dilutions on Columbia sheep blood agar plates (Oxoid) and counting c.f.u. of α-haemolytic streptococci. Survival of *S. suis* in the blood of the piglets was determined by a bactericidal assay conducted as previously described [12] with the following slight modification: 500 µl heparinized blood was infected with 3×10^5^ c.f.u. of exponentially grown bacteria. Bactericidal assays were performed twice, namely with blood samples drawn immediately before piglet No. 346 and the other piglets were experimentally infected at an age of 64 and 67 days.

At necropsy, samples were collected for histological (h) and bacteriological (b) examination as previously described [12]: both carpal and tarsal joints (h, b), cerebrospinal fluid (b), and inner organs such as liver (h, b), spleen (h, b) and lung (h, b). Swabs (b) and further tissue samples (h) were taken from pleura, pericardium, peritoneum, mitral valve and brain. Gross examination revealed pathological findings in the mitral valve, the brain and the spleen (see below). The cerebrospinal fluid was turbid and generated bubbles.

Part of the mitral valve was embedded in methacrylate and submitted to fluorescence *in situ* hybridization (FISH) as described, using panbacterial probe EUB338-Cy5 combined with *Streptococcus*-specific probe STREP1-Cy3 and nucleic acid stain DAPI (4′,6-diamidino-2-phenylindole) [14].

## Diagnosis

**Fig. 1.**
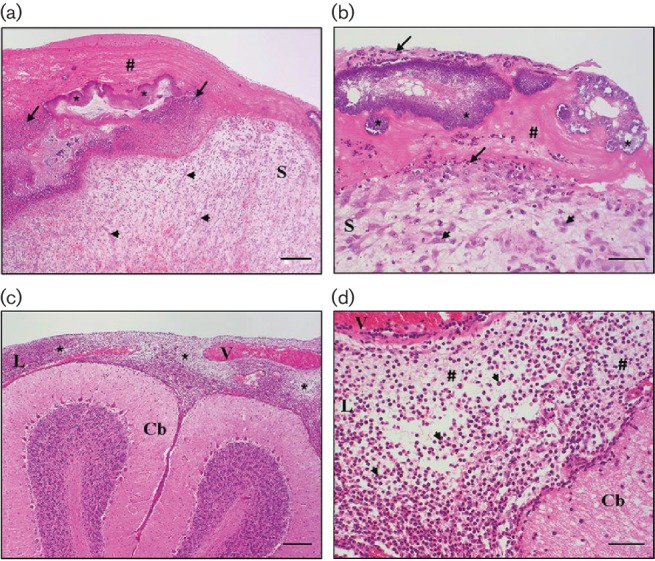
(a, b) Heart, mitral valve, *endocarditis valvularis thromboticans*, polyphasic: the valve leaflet is characterized by a loss of endothelial cells and deposition of fibrin (thrombus #), infiltrated by inflammatory cells (arrows) and with detection of numerous bacterial colonies (coccoid bacteria,*). Within the valvular spongiosa (S), fibroangioblastic granulation tissue (arrowheads) is visible. (c, d) Brain, cerebellum (Cb), fibrinosuppurative leptomeningitis: the leptomeninges (L) are distinctively expanded by a deposition of fibrin and infiltration by neutrophilic granulocytes (*); hyperaemic vessels (V). All panels are haematoxylin and eosin (H.E.)-stained. Bars, 200 µm (a, c), 50 µm (b, d).

The atrial side of the mitral valve showed a moderate to severe polyphasic *endocarditis valvularis thromboticans*. This inflammation was macroscopically characterized by several light to dark brown, firm nodules (diameter 0.3–0.5 cm) with a rough surface. Histomorphologically, these nodules could be identified as thrombotic material deposited at the valve due to loss of the endocardial endothelium. Within the fibrinous network of the thrombi, numerous bacterial colonies consisting of coccoid bacteria as well as an infiltration with neutrophilic, often degenerative, granulocytes were observable (Fig. 1a, b). Below these areas and in the direction of the endothelial defect, the valvular spongiosa showed a distinctive development of fibroangioblastic granulation tissue (Fig. 1a, b).

**Fig. 2.**
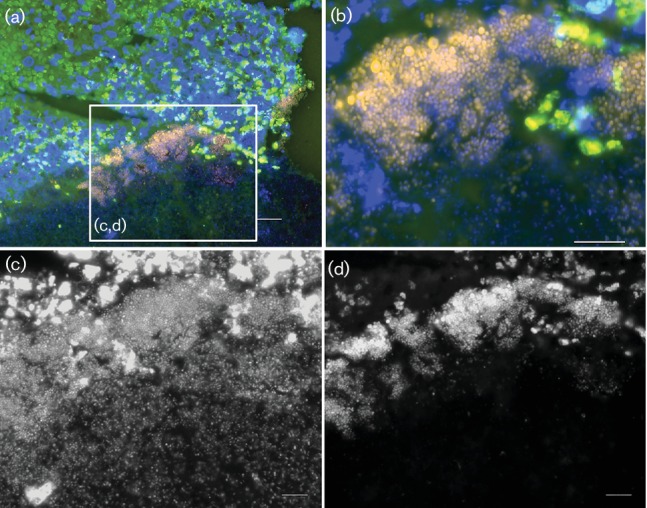
Heart, mitral valve: FISH using *Streptococcus*-specific probe STREP1-Cy3 (orange) and nucleic acid stain DAPI (blue) shows a rich and structured biofilm. (a) Overview of the FISH-positive biofilm against the green background fluorescence of the tissue; (b) at higher resolution single FISH-positive cocci are visible. Note the discriminative fluorescence intensity in orange as compared to the DAPI signal indicating differential ribosomal content of the bacteria. (c, d) Insert at higher magnification showing the identical microscopic field in greyscale with separate microscopic channels. Whereas DAPI shows numerous cocci scattered throughout the tissue (c), the *Streptococcus*-FISH probe detects mainly the upper layer of bacteria with the strongest signals at the top of the biofilm (d). Bars, 20 µm (a), 10 µm (b, c, d).

FISH of the mitral valve showed extensive and stratified biofilms (Fig. 2). All bacteria detected by panbacterial probe EUB338 were also detected by *Streptococcus*-specific probe proving a monospecies infection (data not shown).

A slight hyperaemia of the meningeal vessels was the only macroscopically noticeable finding of the brain. However, by histomorphological investigation a diffuse, moderate to severe, fibrinosuppurative leptomeningitis was diagnosed (Fig. 1c, d), also expanding to the perivascular Virchow-Robin spaces. In addition to neutrophilic granulocytes, macrophages infiltrated the tissue.

The spleen showed a follicular hyperplasia, indicating an immunoreactive response. Moreover, a mild, suppurative splenitis was found by light microscopy.

Measurement of IgG antibody titres against the vaccine antigen rIde*_Ssuis_* revealed that the piglet seroconverted to a positive titre upon vaccination (0.7 to 45.3). IgG antibodies against MRP were not detected prior the experimental infection but were detected 11 days post-infection (45 ELISA units).

Bactericidal assays showed that *S. suis* strain 10 (*cps*2) and the challenge strain A3286/94 (*cps*9) were killed in the blood of piglet No.346 *ex vivo* prior to both experimental infections with bacterial survival rates of 0 to 0.09 (*cps*2) and 0 to 0.04 (*cps*9) compared to 4.46 to 4.68 (*cps*2) and 1.14 to 1.16 (*cps*9) in the placebo-treated piglets.

Through culture and PCR-based differentiation of α-haemolytic streptococci [3], the challenge strain was detected in the cerebrospinal fluid, brain, spleen and mitral valve.

## Discussion

Endocarditis is a main manifestation of *S. suis* infection in piglets [15–17] and has also been recorded numerously in humans [5, 18–20] infected with this pathogen. Here, we describe a case of an experimentally induced severe polyphasic *endocarditis valvularis thromboticans* in a piglet with substantial specific bactericidal activity against *S. suis* in the blood. The pathogenesis of this endocarditis was most likely associated with biofilm formation as coccoid bacteria were detectable in the fibrinous network covering the mitral valve. Using FISH combined with DAPI stain, the biofilms showed a stratified pattern with FISH highly positive layers. Since FISH probes target 16S rRNA, the signal intensity correlates to the ribosome content, indicative that the cells were active (presumably viable) at time of fixation [21, 22]. The polyphasic (chronically active) character of this lesion indicates that this pathology started early after experimental infection. Note, that experimental infection elicited additional antibodies directed against surface antigens such as MRP. However, bacteria buried in the vegetation were obviously not killed efficiently.

Apart from a fluctuating inner body temperature, the piglet was clinically unobtrusive for 10 days post-infection. It is reasonable to hypothesize that the pathogenesis of endocarditis progressed during this time. The clinical presentation changed abruptly on the 11th day post-infection as the piglet demonstrated clinical signs of severe meningitis, namely neck stiffness with pain vocalization. Based on the acute character of the histologically confirmed fibrinosuppurative leptomeningitis, it is most likely a complication of the polyphasic *endocarditis valvularis thromboticans*. Accordingly, Karstrup *et al*. [15] describe association of *S. suis* endocarditis with focal suppurative encephalitis and leptomeningitis in slaughter pigs. In the case reported here, the leptomeningitis was not locally restricted but diffuse, thus explaining the rapid clinical deterioration. Though we did not record septic embolism, this appears to be a likely cause of the leptomeningitis.

Biofilm formation is a known feature of *S. suis* and its extent depends on the serotype. Serotype 9 was shown to be the most potent one [23]. As coccoid bacteria were identified in a deposition of fibrin, it seems likely that fibrinogen, which is known to induce biofilm formation by *S. suis* [24], played a role in this process. Since the function of MRP was recently uncovered as being a fibrinogen-binding protein [25], it might also contribute to this trait. The prominent formation of biofilms by this serotype on different tissues including the mitral valve might be related to a low protective efficacy of *S. suis* serotype 9 bacterins in autogenous vaccination of respective herds. Accordingly, endocarditis has been recorded previously in bacterin-vaccinated piglets challenged with *S. suis* serotype 9 [11]. Noteworthy, this case report does not exclude protection of the vaccination antigen against *S. suis* serotype 9 as rapid onset of disease, most likely independent of biofilm formation, has also been observed following challenge with this pathotype [11].

We hypothesize that the described complication might also occur in humans, as *S. suis* is also a cause of endocarditis in humans. Since meningitis induced by *S. suis* infection is very often severe and associated with permanent hearing loss in humans [26], clinicians seeing endocarditis patients should be aware of this pathogen.
